# Photo Quiz: No brainer—hydrocephalus in a 39-year-old male

**DOI:** 10.1128/jcm.01248-25

**Published:** 2026-05-05

**Authors:** David Barrera, Arthur H. Totten, Jared K. Woods

**Affiliations:** 1 Department of Pathology, Renaissance School of Medicine, Stony Brook University 200793, Stony Brook, New York, USA; Mayo Clinic Minnesota, Rochester, Minnesota, USA

## PHOTO QUIZ 

**Fig 1:**
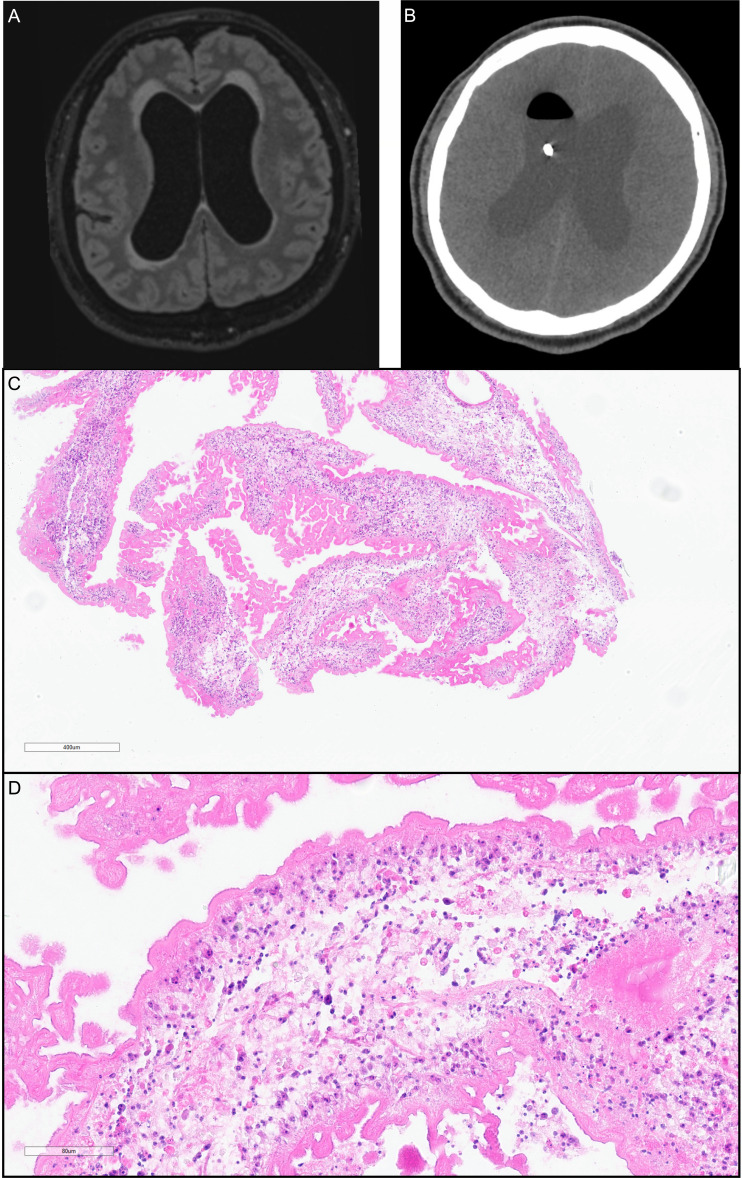
Patient scan and histologic findings. (**A**) Magnetic resonance imaging (MRI) and (**B**) computed tomography (CT) without contrast of patient pre- (**A**) and post-ventriculostomy (**B**) catheter insertion. Dilated lateral ventricles are seen on both images. A hyperintense catheter is seen on CT imaging. (**C**) Hematoxylin-and-eosin (H&E) stain of brain biopsy specimen of fourth ventricle cystic lesion at low power (6×) and high power (40×, **D**).

A 39-year-old El Salvadorian male with unknown recent travel history presented to the emergency room with daily holocephalic headaches. Pain was more prominent on the left side, and the patient had bilateral transient vision loss for the past 3 months, where he was found to have worsening hydrocephalus on magnetic resonance imaging (MRI) and on computed tomography (CT) imaging (Fig. 1A and B). The patient stated his symptoms, including constant headaches, started after a motor vehicle accident 2 years prior to his current presentation. CT imaging at initial presentation showed hydrocephalus, but he was lost to follow-up until current presentation. Laboratory findings at the time of current presentation were significant for relative eosinophilia (9.4%, normal 0–3%) with no absolute eosinophilia and normal white blood count (8.41 K/µL, normal 4.80–10.80 K/µL). Cerebrospinal fluid (CSF) was clear and colorless with an elevated cell count of 16 nucleated cells (normal range 0–5 cells/µL) and high glucose (76 mg/dL, normal 40–70 mg/dL). The patient was brought to the operating room for an endoscopic third ventriculocisternostomy, which revealed an opening pressure greater than 20 cm of water, a thickened portion of the floor of the third ventricle that contained a free-floating sizable cystic lesion, and multiple small cysts in the fourth ventricle that appeared as “a cluster of grapes” during the procedure. A CSF specimen was collected and sent for bacterial and mycobacterial (AFB) cultures, which showed no growth. Stool ova-and-parasite stain was negative for pathogens. A tissue biopsy was sent for histologic evaluation (Fig. 1C and D).

What is your diagnosis?

## ANSWER TO PHOTO QUIZ

*Taenia solium* cysts identified in the ventricular space are compatible with racemose neurocysticercosis (NCC). Hematoxylin-and-eosin (H&E) stains of brain cyst biopsy reveal a three-layered cysticercus with an outermost, bright pink cuticular layer which demonstrates surface infoldings that are lined with microtriches consistent with the outer layer of tegument membrane (Fig. 1C and D) (1, 2). The cellular layer underneath the cuticular layer shows a syncytium of cell bodies with uniform nuclei (1). Finally, the deepest layer known as the inner reticular layer consists of loosely organized stroma (1). A scolex was not identified in the tissue examined. The absence of scolices within the cysticercus wall is supportive of a racemose cyst caused by *T. solium* (2, 3). A differential of cyst forms in the brain includes coenurosis, which demonstrates numerous protoscoleces and cystic echinococcus hydatid cyst, which consists of two layers (4, 5).

Neurocysticercosis is the most common helminthic infection of the central nervous system, especially in countries of low socioeconomic development (3). Parenchymal and extraparenchymal cysts may occur from the ingestion of eggs or gravid proglottids from the feces of a definitive host (3). The larval form of *T. solium* is the developmental stage that leads to cysticerci formation, which may cause symptoms of headache and seizure in NCC (3). Racemose NCC is considered a more aggressive form of NCC and is seen in 15–54% of patients (3). Extraparenchymal racemose NCC can demonstrate cystic or grape-like structures within the brain’s ventricular system, which can subsequently cause intracranial hypertension. The subarachnoid form of racemose NCC can lead to arteritis and stroke (2, 5, 6). The meningeal form of racemose NCC can lead to hydrocephalus and meningitis. Those who present with hydrocephalus secondary to NCC meningitis have a 50% mortality rate (3). However, those who have hydrocephalus requiring a ventriculoperitoneal shunt, as in this patient, have a mortality rate closer to 20% due to complications, while overall mortality for all forms of NCC is 5% (7, 8).

Humans can serve as both a definitive and intermediate host, and individuals who consume raw or undercooked meat containing larvae can develop taeniasis (9). Ingestion of contaminated food or water containing *T. solium* eggs can lead to NCC (10, 11). Careful consideration of clinical history, as well as travel to endemic areas and exposure history, is paramount in considering NCC as a diagnosis.
